# Supplementary material for: MINT: a multivariate integrative method to identify reproducible molecular signatures across independent experiments and platforms
Source: BMC Bioinformatics. 2017 Feb 27;18:128. doi: 10.1186/s12859-017-1553-8 (PMC5327533; doi:10.1186/s12859-017-1553-8)
Supplement: Supplementary file 1 — Supplementary material. This pdf document contains supplementary methods and all supplementary Figures and Tables. Specifically, it provides the PLS-algorithm, the extension of MINT in a regression framework, the application to the MAQC data (A vs B), the meta-analysis of the breast cancer data, the classification accuracy of the tested methods on the stem cells and breast cancer data, and details on the signature genes identified by MINT on the stem cells and breast cancer data. (PDF 4403 kb) [file 12859_2017_1553_MOESM1_ESM.pdf]

SUPPLEMENTARY MATERIAL for: MINT: A multivariate integrative method to identify reproducible molecular signatures across independent experiments and platforms.

F. Rohart [1], A. Eslami [2], N. Matigian [1], S. Bougeard [3], K-A. Lê Cao [1,*]

[1]The University of Queensland Diamantina Institute,
The University of Queensland, Translational Research Institute, QLD 4102, Australia,
[2] Faculty of Medicine, University of British Columbia, Vancouver, Canada and
[3]French agency for food, environmental and occupational health safety (Anses),
Department of Epidemiology, 22440 Ploufragan, France

Contents

S1 PLS-algorithm 3

S2 Extension of MINT for a regression framework 4

S3 Application to the MAQC data 6
- S3.1 A vs B 6
- S3.2 C vs D 7

S4 Limitations of common meta-analysis and integrative approaches - breast cancer study 8

S5 *MINT* outperforms state-of-the-art methods 9

S6 Application to the stem cells data 12
- S6.1 Meta-analysis 12
- S6.2 Signature identified by MINT 13
- S6.3 Study-specific output of MINT 14

S7 Application to the breast cancer data 15

S8 OCT4 expression 17

List of Figures

S1 Experimental design of *MINT*, combining M independent studies $X^{(m)}, Y^{(m)}$, where $X^{(m)}$ is a data matrix of size n_m observations (rows) $\times$ P variables (e.g. gene expression levels, in columns) and $Y^{(m)}$ is a dummy matrix indicating each sample class membership of size n_m observations (rows) $\times$ K categories outcome (columns). 4

S2 Workflow of the *MINT* algorithm. Black lines represent matrix multiplication; orange dashed lines represent addition and purple dotted lines represent no operation. 5

S3 MAQC data, A vs B. (left) Venn diagram showing the overlap of differential expressed genes with a FDR < 0.0001 among the three platforms and (right) Principal Component Analysis highlighting a batch effect in the MAQC data. 7

S4 MAQC data, C vs D. (left) Venn diagram showing the overlap of differential expressed genes with a FDR < 0.0001 among the three platforms and right Principal Component Analysis highlighting a batch effect in the MAQC data C vs D. 8

S5 Breast cancer data. (Top) Venn Diagram of the genes declared as Differentially Expressed with ANOVA (FDR$< 10^{-6}$), (bottom left) Principal Component Analysis and (bottom right) PLSDA on the concatenated data for the three independent training sets of breast cancer data referenced in Table 2. . . 9
S6 Stem cells data. Balanced Error Rate (BER), the lower the error rate the better the classification performance, top) and classification error rate per class (the higher the classification accuracy the better, bottom), for both training and independent test set. 10
S7 Breast cancer data. Balanced Error Rate (BER), the lower the error rate the better the classification performance, top) and classification error rate per class (the higher the classification accuracy the better, bottom), for both training and independent test set. 11
S8 Average rank of Balanced Error Rate (BER) performance for the four evaluations of training and test sets for the stem cells and breast cancer studies. Sixteen combination of statistical methods are plotted as well as the *MINT* approach, which is highlighted in black. 12
S9 Study-specific results of *MINT* for the three cell types of eight studies referenced in Table 1. *MINT* model was obtained with two and fifteen genes on the first two component, respectively (Table S3). . . 14
S10 Breast cancer data. Boxplot of the standardised gene expression for the 11 genes identified by MINT (data is centered and scaled per study), for each of the four subtypes of breast cancer (Table 2). . . . 17
S11 OCT4 gene expression, stem cells data. (First row) YuGene-normalized gene expression and (second row) MINT-standardised gene expression (data is centered and scaled per study) of OCT4 compared to the two genes, LIN28A and CAR, selected by *MINT* on the first component. P-value from a t-test of Fibroblasts vs both hESC and hiPSC is provided. 18
S12 OCT4 gene expression for each study of the stem cells data. (First row) YuGene-normalized gene expression and (second row) MINT-standardised gene expression (data is centered and scaled per study) of OCT4 compared to the two genes, LIN28A and CAR, selected by *MINT* on the first component. P-value from a t-test of Fibroblasts vs both hESC and hiPSC is provided. 19

List of Tables

S1 Experimental design of the MAQC data, with four biological samples A, B, C and D. 6
S2 Meta-analysis of the stem cells data. Genes commonly declared as Differentially Expressed with a FDR$< 10^{-5}$ for the human cell types of eight studies referenced in Table 1 12
S3 Genes selected by *MINT* on two components for the eight independent studies of the stem cells data referenced in Table 1. 13
S4 Genes selected by *MINT* on the first component tuned by LOGOCV, for the three independent training sets of the breast cancer data (including PAM50 genes) referenced in Table 2. 15
S5 Genes selected by *MINT* on the first three components tuned by LOGOCV, for the three independent training sets of the breast cancer data (excluding PAM50 genes) referenced in Table 2. 16

S1 PLS-algorithm

Let X and Y be high dimensional (quantitative) data matrices of size $N \times P$ and $N \times Q$, respectively. These matrices can be high-dimensional. PLS regression is an iterative method that constructs successive artificial components $t_h = X_h a_h$ and $u_h = Y_h b_h$ for $h = 1, .., H$, where the hth-PLS component t_h (respectively u_h) is a linear combination of X's variables (Y's), in which the vector of weights a_h (b_h) is called loading vector. The PLS-algorithm is described next in a context of regression of Y onto X.

Algorithm 1 *PLS*

Denote $X_1 = X$ and $Y_1 = Y$..
For h in $1, \dots, H$, choose an initial value for a_h with $||a_h||_2 = 1$
repeat
  $t_h \leftarrow X_h a_h$ ▷ PLS component for X
  $b_h \leftarrow (Y_h)^\top t_h$ ▷ loading vector for X
  $b_h \leftarrow b_h / ||b_h||_2$ ▷ Standardisation
  $u_h \leftarrow Y_h b_h$ ▷ PLS component for Y
  $a_h \leftarrow (X_h)^\top u_h$ ▷ loading vector for Y
  $a_h \leftarrow a_h / ||a_h||_2$ ▷ standardisation
until convergence of a_h and b_h
$P \leftarrow I - t_h (t_h^\top t_h)^{-1} t_h^\top$, I = identity matrix of $\mathbb{R}^N$
$X_{h+1} \leftarrow P X_h$ and $Y_{h+1} \leftarrow P Y_h$ ▷ deflation

S2 Extension of MINT for a regression framework

We assume that the data are partitioned into M groups corresponding to each independent study m: $\{(X^{(1)}, Y^{(1)}), \ldots, (X^{(M)}, Y^{(M)})\}$ so that $\sum_{m=1}^{M} n_m = N$, where n_m is the number of samples in group m, see Figure S1 for a classification framework. Each variable from the data set $X^{(m)}$ and $Y^{(m)}$ is centered and has unit variance. We write X and Y the concatenation of all $X^{(m)}$ and $Y^{(m)}$, respectively.

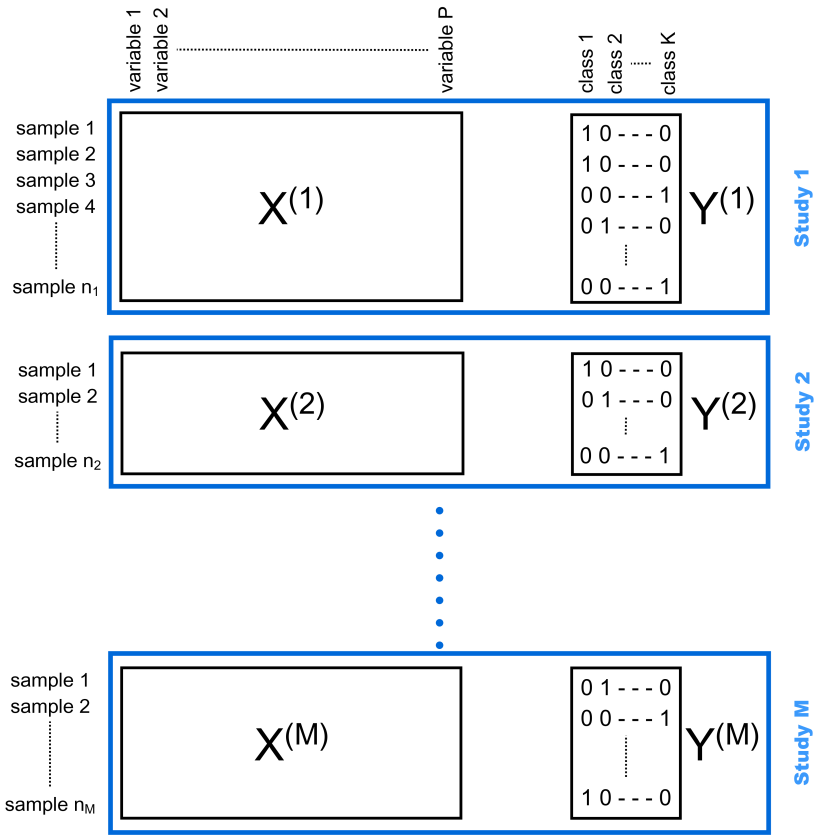

Figure S1: Experimental design of *MINT*, combining M independent studies $X^{(m)}, Y^{(m)}$, where $X^{(m)}$ is a data matrix of size n_m observations (rows) $\times$ P variables (e.g. gene expression levels, in columns) and $Y^{(m)}$ is a dummy matrix indicating each sample class membership of size n_m observations (rows) $\times$ K categories outcome (columns).

Previously, for a classification framework, Y was a dummy matrix indicating the class membership of each sample. We adapted the *MINT* algorithm to the regression framework in which Y is a quantitative data matrix. The *MINT* regression applies for example when modelling a multiple multivariate regression between transcriptomics data sets (X) and clinical parameters (Y).

We added a regularisation parameter λ_2 to select variables of Y through a ℓ^1 penalisation. In this case, the function to maximize is

$$\max_{||a_h||_2=||b_h||_2=1} \sum_{m=1}^{M} n_m cov(X_h^{(m)} a_h, Y_h^{(m)} b_h) + \lambda_1 ||a_h||_1 + \lambda_2 ||b_h||_1, h = 1, \ldots, H,$$

where in addition to (3), λ_2 is a non negative parameter that controls the amount of shrinkage and thus the number of non zero weights in the global loading vectors b_h. Both global loadings vectors a_h and b_h can be seen on the workflow of the *MINT* approached (Figure S2). The *MINT* extension addresses simultaneously

aims (1) and (3); it integrates different experiments while selecting the most relevant variables. The *MINT* pseudo algorithm in the context of regression in as follows.

Algorithm 2 *MINT* for regression

Denote $\forall 1 \leq m \leq M, X_1^{(m)} = X^{(m)}$, $Y_1^{(m)} = Y^{(m)}$, $X^{(m)} = X$ and $Y^{(m)} = Y$.
For h in $1, \ldots, H$, choose λ_h, γ_h and an initial value for a_h with $||a_h||_2 = 1$
repeat
  $t_h^{(m)} \leftarrow X_h^{(m)} a_h$ ▷ partial components
  $t_h \leftarrow X_h a_h$ ▷ global components
  $b_h^{(m)} \leftarrow (Y_h^{(m)})^\top t_h^{(m)}$ ▷ partial loadings
  $b_h \leftarrow (\sum_{m=1}^{M} b_h^{(m)})/|| \sum_{m=1}^{M} b_h^{(m)}||_2$ ▷ global loadings
  $b_h \leftarrow sign(b_h)(|b_h| - \gamma_h)_+$ ▷ soft thresholding
  $u_h^{(m)} \leftarrow Y_h^{(m)} b_h$ ▷ partial components
  $a_h^{(m)} \leftarrow (X_h^{(m)})^\top u_h^{(m)}$ ▷ partial loadings
  $a_h \leftarrow (\sum_{m=1}^{M} a_h^{(m)})/|| \sum_{m=1}^{M} a_h^{(m)}||_2$ ▷ global loadings
  $a_h \leftarrow sign(a_h)(|a_h| - \lambda_h)_+$ ▷ soft thresholding
until convergence of a_h and b_h
$P \leftarrow I - t_h(t_h^\top t_h)^{-1} t_h^\top$, $I =$ identity matrix of $\mathbb{R}^N$
$X_{h+1} \leftarrow PX_h$ and $Y_{h+1} \leftarrow PY_h$ ▷ deflation

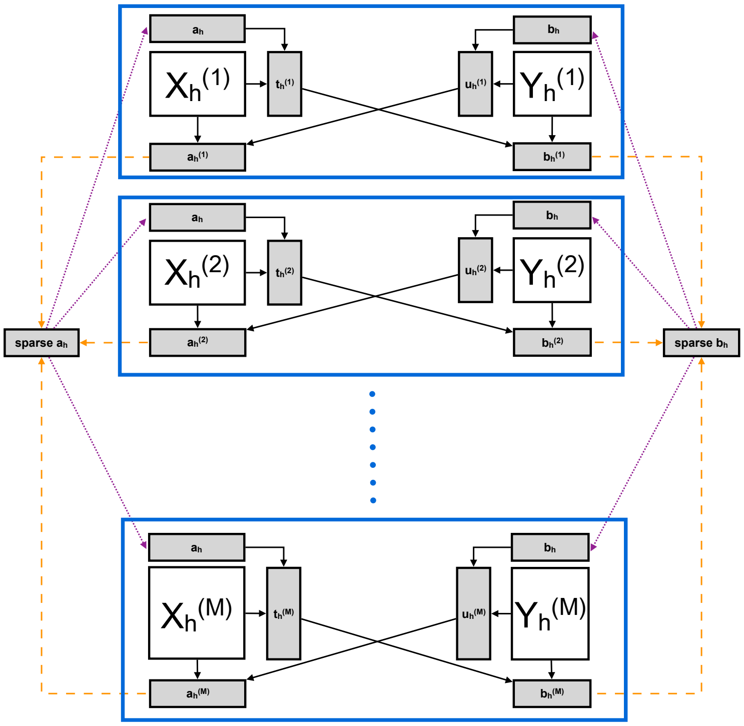

Figure S2: Workflow of the *MINT* algorithm. Black lines represent matrix multiplication; orange dashed lines represent addition and purple dotted lines represent no operation.

Choosing the regularization parameters λ_h and γ_h for each of the H PLS-components can be achieved

through Cross-Validation (CV). Note that this tuning step can be computationally intensive if feature selection must be achieved on both X and Y data sets concurrently. Computational cost of CV is reduced when tuning a single parameter, as in a classification framework or when X or Y is univariate.

S3 Application to the MAQC data

Platform	A	B	C	D
Illumina HT-12	6	6	6	6
Affy HU GeneST 2.0	4	4	4	4
Affy Primeview	4	4	4	4
3 platforms	14	14	14	14

Table S1: Experimental design of the MAQC data, with four biological samples A, B, C and D.

S3.1 A vs B

A differential expression analysis of A vs B was conducted on each of the three microarray platforms using ANOVA, and revealed an overlap among the platforms of 2717 DEG with a False Discovery Rate of 10^{-4} (Benjamini and Hochberg, 1995). This corresponds to 59.6% of all DEG for illumina, 47.3% for Affymetrix HuGene and 36.5% for Affymetrix Prime (Figure S3). We observed that conducting a differential analysis on the concatenated data from the three microarray platforms without accomodating for batch effects resulted in 2460 DEG, of which only 61.4%(1669) were part of the common 2717 DEG. This implies that 38.6% (791) of these genes were not found DE with a FDR of 10^{-4} in at least one study. Thus, the biological effect is most likely confounding with the technical effect for these 791 false positive genes. A Principal Component Analysis (PCA) sample plot confirmed that the major source of variation in the combined data is attributed to platforms as the samples clustered by platforms rather than by outcome class (see Figure S3).

Using LOGOCV to choose the optimal number of genes to discriminate the two biological classes with *MINT* on one component, a single gene "CKS2" was selected. CKS2 was also part of the common list of DEG and was ranked 2 for illumina, 3 for affyPrime and 362 for affyHugene. Since the biological samples to discriminate are very different, it was not surprising that *MINT* only selected one gene to achieve the best classification accuracy. However, to further compare the results of our approach to the 2717 common DEG, we manually required *MINT* to select more genes via the 'keepX' argument that was implemented in the mixOmics package to control the amount of sparsity. A very high overlap was reported between the genes selected by *MINT* and the 2717 common genes that are assumed to be true positive; when MINT was ask to select 500 genes, 100% of these were found in the common genes, this percentage remained high at 84.8% when *MINT* was asked to select 2717 genes.

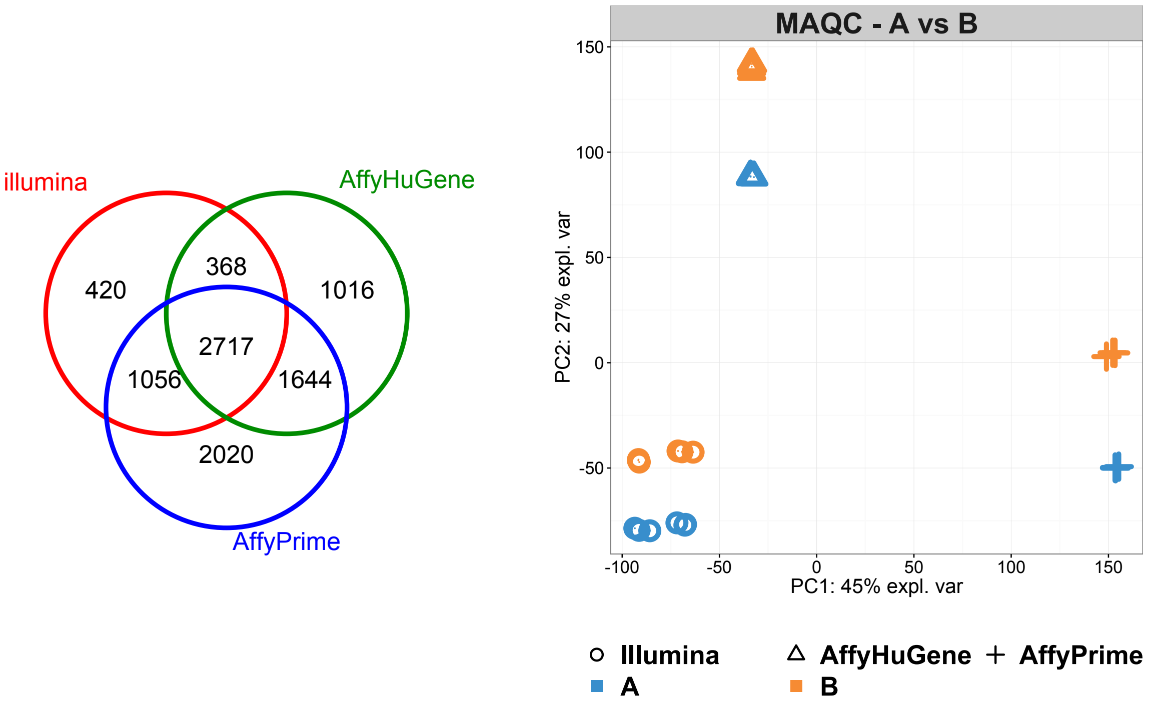

Figure S3: MAQC data, A vs B. (left) Venn diagram showing the overlap of differential expressed genes with a FDR < 0.0001 among the three platforms and (right) Principal Component Analysis highlighting a batch effect in the MAQC data.

S3.2 C vs D

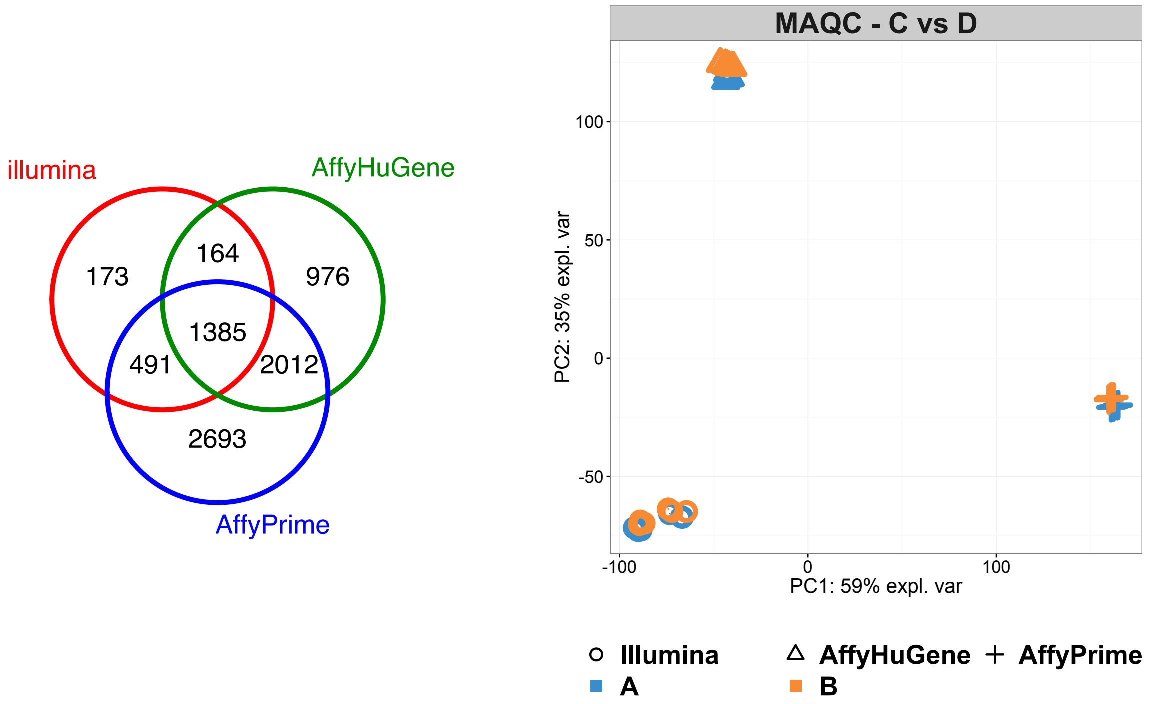

Figure S4: MAQC data, C vs D. (left) Venn diagram showing the overlap of differential expressed genes with a FDR < 0.0001 among the three platforms and right Principal Component Analysis highlighting a batch effect in the MAQC data C vs D.

S4 Limitations of common meta-analysis and integrative approaches - breast cancer study

Similarly to the the analysis of the human cell types data in Section 2 of the manuscript, we illustrate the shortcomings of (i) a classical meta-analysis and (ii) an integrative analysis using the three independent training datasets described in Table 2. First, a PCA sample plot representation illustrates the need to accommodate for unwanted variation as both METABRIC studies are clustering together but away from the TCGA RNA-seq experiment (Figure S5B). We observed that the unwanted variation accounted for 75% of the total variability of the data. For (i), a DE analysis was performed with ANOVA (FDR $< 10^{-6}$). The Venn-Diagram depicted in Figure S5 highlights a high concordance of DEG between the METABRIC discovery and validation sets but a low concordance between METABRIC and TCGA. The low concordance between METABRIC sets and TCGA is most likely due to the use of a difference commercial platform; conversely, both METABRIC sets used the same platform. Concerning (ii), PLS-DA was not able to discriminate any of the four subtypes of breast cancer, although a diagonal trend can be seen from bottom right with *Basal* samples, to top left with *Luminal* samples (Figure S5C). The latter result highlight the limitation of integrative analysis in this challenging analytical context.

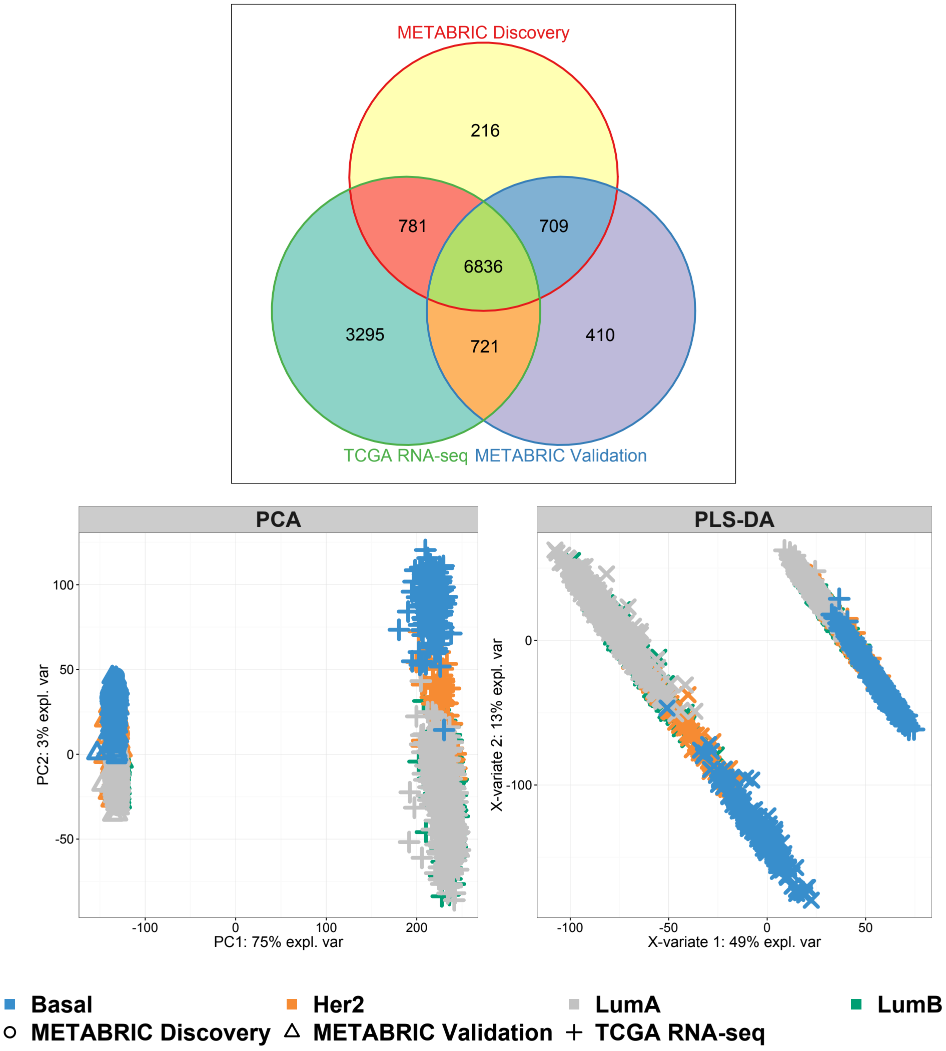

Figure S5: Breast cancer data. (Top) Venn Diagram of the genes declared as Differentially Expressed with ANOVA (FDR$< 10^{-6}$), (bottom left) Principal Component Analysis and (bottom right) PLSDA on the concatenated data for the three independent training sets of breast cancer data referenced in Table 2.

S5 *MINT* outperforms state-of-the-art methods

Details on the methods are available in the manuscript and results are provided in Figure S6 for the stem cells data and Figure S7 for the analysis of the breast cancer data in which the PAM50 genes were removed. The classification accuracy results were similar when the PAM50 genes were included (not shown).

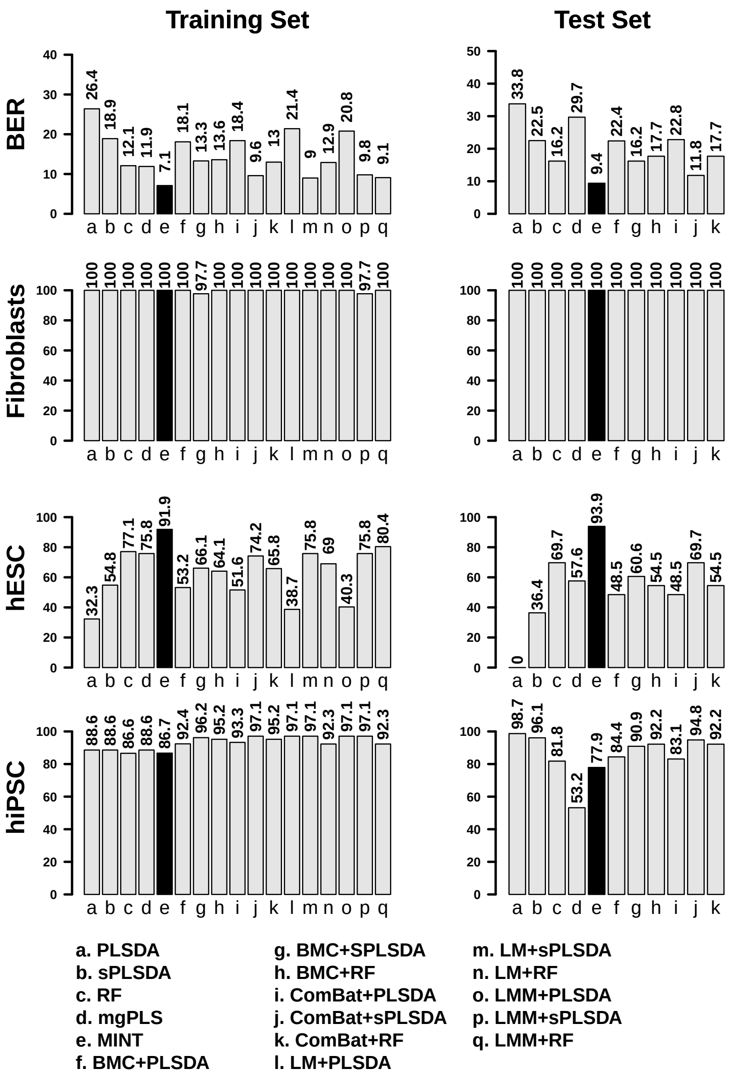

Figure S6: Stem cells data. Balanced Error Rate (BER), the lower the error rate the better the classification performance, top) and classification error rate per class (the higher the classification accuracy the better, bottom), for both training and independent test set.

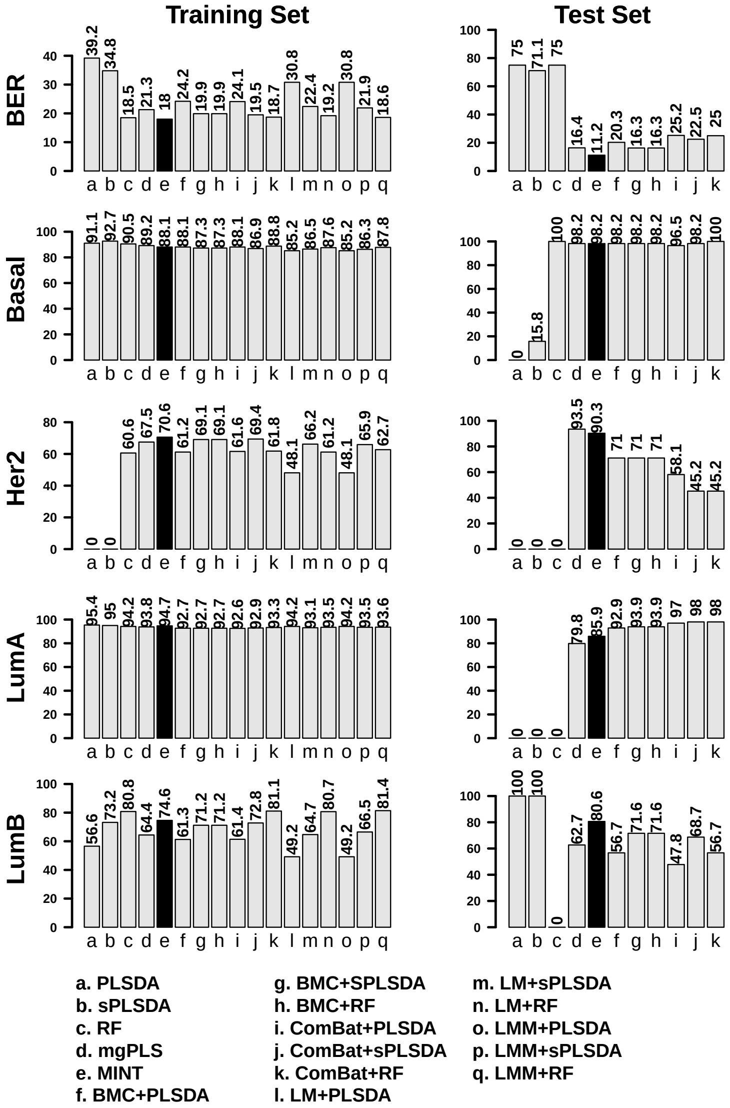

Figure S7: Breast cancer data. Balanced Error Rate (BER), the lower the error rate the better the classification performance, top) and classification error rate per class (the higher the classification accuracy the better, bottom), for both training and independent test set.

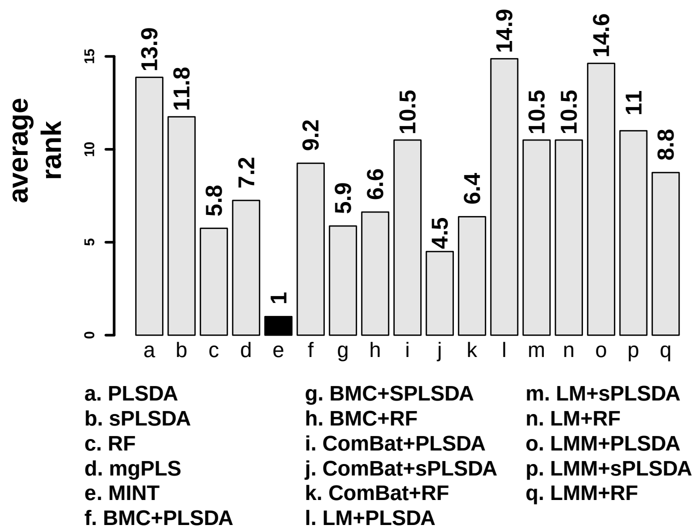

Figure S8: Average rank of Balanced Error Rate (BER) performance for the four evaluations of training and test sets for the stem cells and breast cancer studies. Sixteen combination of statistical methods are plotted as well as the *MINT* approach, which is highlighted in black.

S6 Application to the stem cells data

S6.1 Meta-analysis

Ensembl ID	Symbol
ENSG00000091972	CD200
ENSG00000092421	SEMA6A
ENSG00000121570	DPPA4
ENSG00000154639	CXADR/CAR
ENSG00000178445	GLDC

Table S2: Meta-analysis of the stem cells data. Genes commonly declared as Differentially Expressed with a FDR$< 10^{-5}$ for the human cell types of eight studies referenced in Table 1

S6.2 Signature identified by MINT

The *MINT* approach was performed with 2 components that were tuned by LOGOCV; 2 and 15 genes were selected on the first two components, respectively (Table S3).

Comp	Ensembl ID	Gene Name	
1	ENSG00000131914	LIN28A	lin-28 homolog A (C. elegans)
1	ENSG00000154639	CXADR/CAR	coxsackie virus and adenovirus receptor
2	ENSG00000137871	ZNF280D	zinc finger protein 280D
2	ENSG00000129317	PUS7L	pseudouridylate synthase 7 homolog (S. cerevisiae)-like
2	ENSG00000155008	APOOL	apolipoprotein O-like
2	ENSG00000095261	PSMD5	proteasome (prosome, macropain) 26S subunit, non-ATPase, 5
2	ENSG00000171466	ZNF562	zinc finger protein 562
2	ENSG00000214553	LRRC37A11P	leucine rich repeat containing 37, member A11, pseudogene
2	ENSG00000149531	FRG1B	FSHD region gene 1 family, member B
2	ENSG00000102226	USP11	ubiquitin specific peptidase 11
2	ENSG00000188372	ZP3	zona pellucida glycoprotein 3 (sperm receptor)
2	ENSG00000243708	PLA2G4B	phospholipase A2, group IVB (cytosolic)
2	ENSG00000123562	MORF4L2	mortality factor 4 like 2
2	ENSG00000220023	AL592183.1	
2	ENSG00000108479	GALK1	galactokinase 1
2	ENSG00000109536	FRG1	FSHD region gene 1
2	ENSG00000137806	NDUFAF1	NADH dehydrogenase (ubiquinone) complex I, assembly factor 1

Table S3: Genes selected by *MINT* on two components for the eight independent studies of the stem cells data referenced in Table 1.

S6.3 Study-specific output of MINT

Figure S9 depicts study-specific graphical outputs for *MINT*. As displayed in Figure 1E of the manuscript, all experiments gave satisfactory classification accuracies for all cell types except Bock and Takahashi studies.

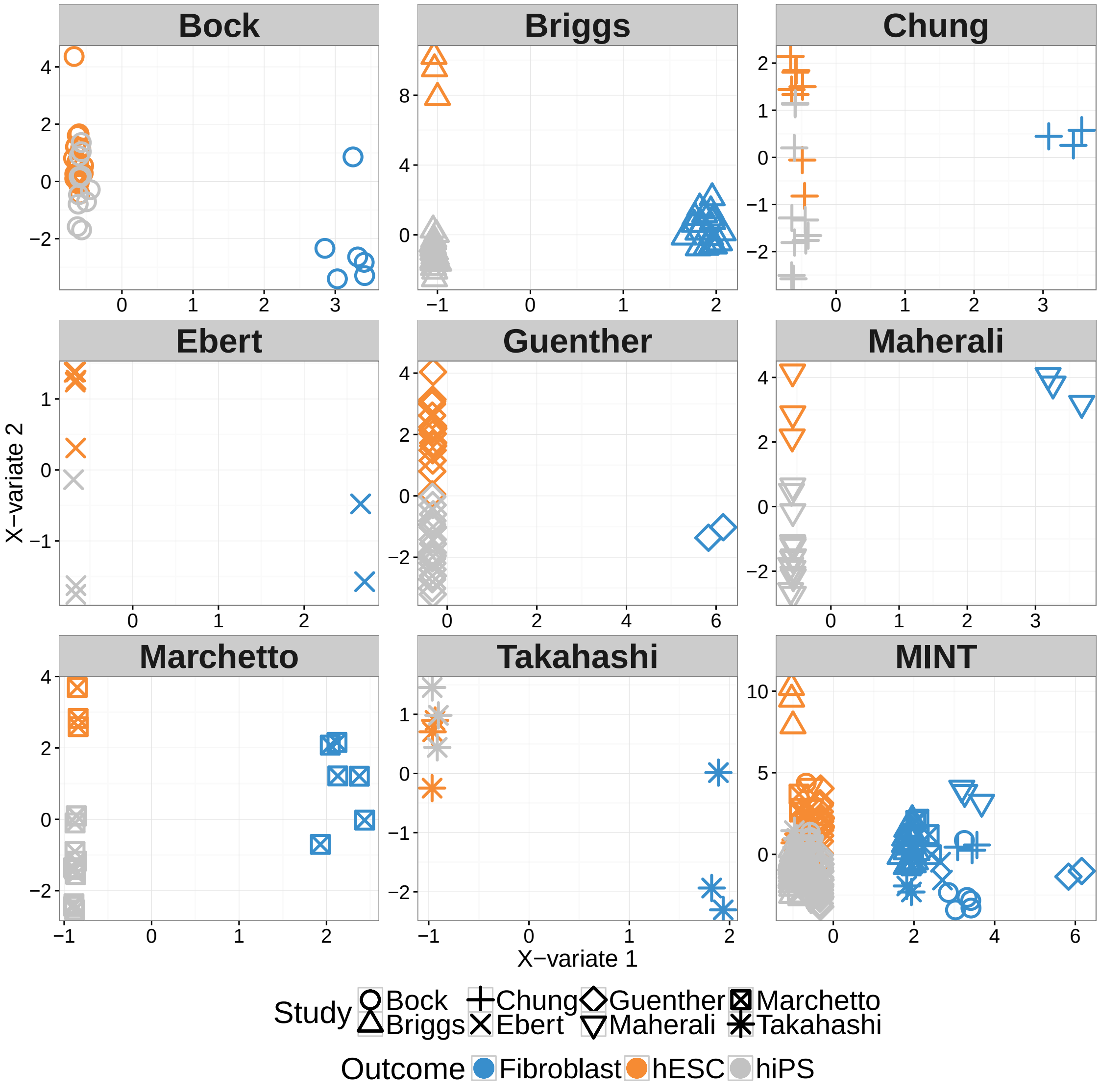

Figure S9: Study-specific results of *MINT* for the three cell types of eight studies referenced in Table 1. *MINT* model was obtained with two and fifteen genes on the first two component, respectively (Table S3).

S7 Application to the breast cancer data

Two *MINT* analyses were performed, with or without the PAM50 genes. In a first analysis (including all genes), *MINT* selected 30, 572 and 636 genes on each three components respectively, using LOGOCV tuning. Genes selected on component 1 are listed in Table S5.

In the second analysis (excluding PAM50 genes), the *MINT* approach selected 11, 272 and 253 genes on the first three components that were tuned by LOGOCV. See Table S5 for a summary of the first component genes. The expression levels of the 11 genes selected on the first component highlight a gradient from *Basal* to *luminal* (Figure S10).

In addition to the details provided in the manuscript, other selected genes in the molecular signature that may have biomarker potential are TBC1D9 (Andres *et al.*, 2013, 2014), DNALI1 (Parris *et al.*, 2010), AFF3 (Lefevre *et al.*, 2015) and CCDC170 (Yamamoto-Ibusuki *et al.*, 2015).

Gene Names	Ensembl ID	Description
AFF3	ENSG00000144218	AF4/FMR2 family, member 3
AGR3	ENSG00000173467	anterior gradient 3 homolog (Xenopus laevis)
ANXA9	ENSG00000143412	annexin A9
ARSG	ENSG00000141337	arylsulfatase G
ARSG	ENSG00000263074	arylsulfatase G
BCL11A	ENSG00000119866	B-cell CLL/lymphoma 11A (zinc finger protein)
C9orf116	ENSG00000160345	chromosome 9 open reading frame 116
CA12	ENSG00000074410	carbonic anhydrase XII
CCDC170	ENSG00000120262	coiled-coil domain containing 170
CCNE1	ENSG00000105173	cyclin E1
CDCA7	ENSG00000144354	cell division cycle associated 7
CMBL	ENSG00000164237	carboxymethylenebutenolidase homolog (Pseudomonas)
DNALI1	ENSG00000163879	dynein, axonemal, light intermediate chain 1
ESR1	ENSG00000091831	estrogen receptor 1
FBP1	ENSG00000165140	fructose-1,6-bisphosphatase 1
FOXA1	ENSG00000129514	forkhead box A1
FOXC1	ENSG00000054598	forkhead box C1
GATA3	ENSG00000107485	GATA binding protein 3
MAPT	ENSG00000186868	microtubule-associated protein tau
MLPH	ENSG00000115648	melanophilin
MYB	ENSG00000118513	v-myb myeloblastosis viral oncogene homolog (avian)
NAT1	ENSG00000171428	N-acetyltransferase 1 (arylamine N-acetyltransferase)
PPP1R14C	ENSG00000198729	protein phosphatase 1, regulatory (inhibitor) subunit 14C
PSAT1	ENSG00000135069	phosphoserine aminotransferase 1
SCUBE2	ENSG00000175356	signal peptide, CUB domain, EGF-like 2
SPDEF	ENSG00000124664	SAM pointed domain containing ets transcription factor
TBC1D9	ENSG00000109436	TBC1 domain family, member 9 (with GRAM domain)
TFF3	ENSG00000160180	trefoil factor 3 (intestinal)
TTLL4	ENSG00000135912	tubulin tyrosine ligase-like family, member 4
VGLL1	ENSG00000102243	vestigial like 1 (Drosophila)
XBP1	ENSG00000100219	X-box binding protein 1

Table S4: Genes selected by *MINT* on the first component tuned by LOGOCV, for the three independent training sets of the breast cancer data (including PAM50 genes) referenced in Table 2.

Gene Names	Ensembl ID	Description
GATA3	ENSG00000107485	GATA binding protein 3
AGR3	ENSG00000173467	anterior gradient 3 homolog (Xenopus laevis)
CA12	ENSG00000074410	carbonic anhydrase XII
TBC1D9	ENSG00000109436	TBC1 domain family, member 9 (with GRAM domain)
PSAT1	ENSG00000135069	phosphoserine aminotransferase 1
XBP1	ENSG00000100219	X-box binding protein 1
TFF3	ENSG00000160180	trefoil factor 3 (intestinal)
DNALI1	ENSG00000163879	dynein, axonemal, light intermediate chain 1
MYB	ENSG00000118513	v-myb myeloblastosis viral oncogene homolog (avian)
CCDC170	ENSG00000120262	coiled-coil domain containing 170
AFF3	ENSG00000144218	AF4/FMR2 family, member 3

Table S5: Genes selected by *MINT* on the first three components tuned by LOGOCV, for the three independent training sets of the breast cancer data (excluding PAM50 genes) referenced in Table 2.

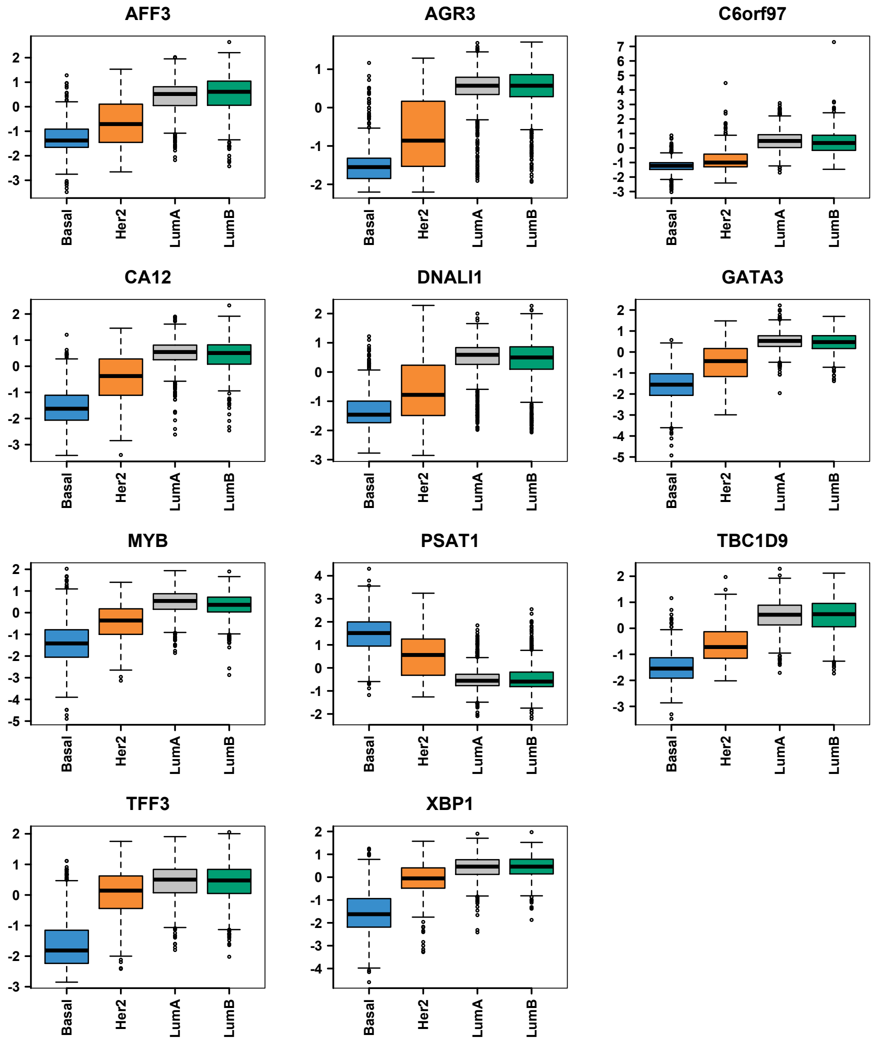

Figure S10: Breast cancer data. Boxplot of the standardised gene expression for the 11 genes identified by MINT (data is centered and scaled per study), for each of the four subtypes of breast cancer (Table 2).

S8 OCT4 expression

OCT4 is the main known marker for undifferentiated cells, we questioned why OCT4 was not selected by *MINT* on the first component. Figure S12 confirms that OCT4 is DE between Fibroblasts and both hESC and hiPSC. However, both LIN28A and CAR are more DE and this was confirmed by a two-sided t-test (Figure S12). We can thus conclude that OCT4 is a discriminant gene, but less informative that LIN28A and CAR, as those seem better suited candidates to discriminate differentiated cells. Further wet laboratory work would be needed to assess the potential and practicality of either LIN28A or CAR. Figure S12 shows the expression of LIN28A, CAR and OCT4 per study and highlights heterogeneity among the studies.

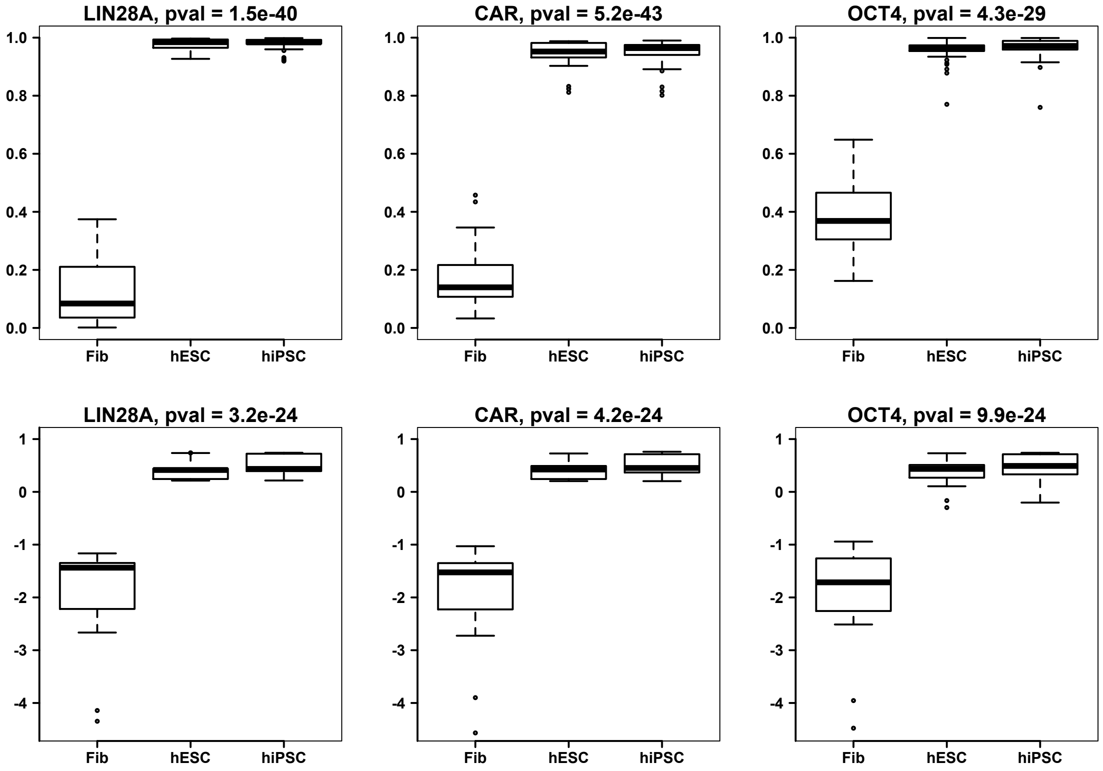

Figure S11: OCT4 gene expression, stem cells data. (First row) YuGene-normalized gene expression and (second row) MINT-standardised gene expression (data is centered and scaled per study) of OCT4 compared to the two genes, LIN28A and CAR, selected by *MINT* on the first component. P-value from a t-test of Fibroblasts vs both hESC and hiPSC is provided.

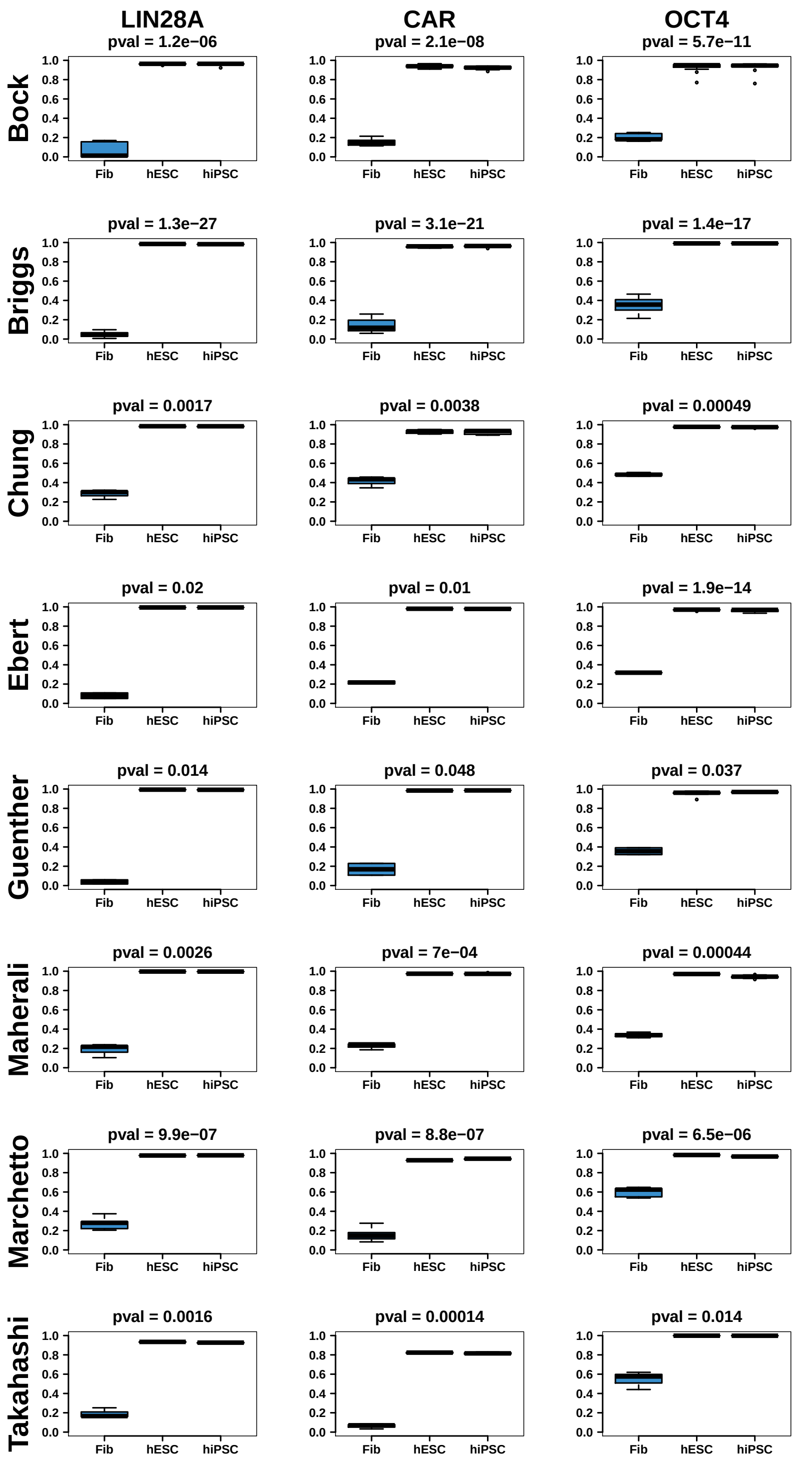

Figure S12: OCT4 gene expression for each study of the stem cells data. (First row) YuGene-normalized gene expression and (second row) MINT-standardised gene expression (data is centered and scaled per study) of OCT4 compared to the two genes, LIN28A and CAR, selected by *MINT* on the first component. P-value from a t-test of Fibroblasts vs both hESC and hiPSC is provided.

References

Andres, S. A., Brock, G. N., and Wittliff, J. L. (2013). Interrogating differences in expression of targeted gene sets to predict breast cancer outcome. *BMC cancer*, **13**(1), 1.

Andres, S. A., Smolenkova, I. A., and Wittliff, J. L. (2014). Gender-associated expression of tumor markers and a small gene set in breast carcinoma. *The Breast*, **23**(3), 226–233.

Benjamini, Y. and Hochberg, Y. (1995). Controlling the false discovery rate: a practical and powerful approach to multiple testing. *J. R. Stat. Soc. Ser. B. Stat. Methodol.*, **57**(1), 289–300.

Lefevre, L., Omeiri, H., Drougat, L., Hantel, C., Giraud, M., Val, P., Rodriguez, S., Perlemoine, K., Blugeon, C., Beuschlein, F., *et al.* (2015). Combined transcriptome studies identify aff3 as a mediator of the oncogenic effects of β-catenin in adrenocortical carcinoma. *Oncogenesis*, **4**(7), e161.

Parris, T. Z., Danielsson, A., Nemes, S., Kovács, A., Delle, U., Fallenius, G., Möllerström, E., Karlsson, P., and Helou, K. (2010). Clinical implications of gene dosage and gene expression patterns in diploid breast carcinoma. *Clin Cancer Res*, **16**(15), 3860–3874.

Yamamoto-Ibusuki, M., Yamamoto, Y., Fujiwara, S., Sueta, A., Yamamoto, S., Hayashi, M., Tomiguchi, M., Takeshita, T., and Iwase, H. (2015). C6orf97-esr1 breast cancer susceptibility locus: influence on progression and survival in breast cancer patients. *Eur J Human Genet*, **23**(7), 949–956.
